# Response of Paroxysmal Nocturnal Hemoglobinuria Clone with Aplastic Anemia to Rituximab

**DOI:** 10.1155/2012/106182

**Published:** 2012-05-07

**Authors:** Radha Raghupathy, Olga Derman

**Affiliations:** ^1^Division of Hematology, Department of Medicine, Montefiore Medical Center, 111E 210th Street, Bronx, NY 10467, USA; ^2^Department of Oncology, Montefiore Medical Center, 111E 210th Street, Bronx, NY 10467, USA

## Abstract

Paroxysmal nocturnal hemoglobinuria is caused by expansion of a hematopoietic stem cell clone with an acquired somatic mutation in the PIG-A gene. This mutation aborts the synthesis and expression of the glycosylphosphatidylinositol anchor proteins CD55 and CD59 on the surface of blood cells, thereby making them more susceptible to complement-mediated damage. A spectrum of disorders occurs in PNH ranging from hemolytic anemia and thrombosis to myelodysplasia, aplastic anemia and, myeloid leukemias. Aplastic anemia is one of the most serious and life-threatening complications of PNH, and a PNH clone is found in almost a third of the cases of aplastic anemia. While allogeneic bone marrow transplantation and T cell immune suppression are effective treatments for aplastic anemia in PNH, these therapies have significant limitations. We report here the first case, to our knowledge, of PNH associated with aplastic anemia treated with the anti-CD20 monoclonal antibody rituximab, which was associated with a significant reduction in the size of the PNH clone and recovery of hematopoiesis. We suggest that this less toxic therapy may have a significant role to play in treatment of PNH associated with aplastic anemia.

## 1. Introduction

Severe aplastic anemia (SAA) is a bone marrow failure syndrome characterized by pancytopenia due to the absence of hematopoietic stem cells in the marrow. SAA is usually fatal, although spontaneous remissions have been documented. Most cases of SAA are idiopathic, but several triggers including drugs, radiation, viral infections, and chemical exposure have been described [1]. Activation of the T cell immune response by these inciting events and subsequent autoimmune destruction of hematopoietic stem cells appears to be pathogenic in most cases of SAA. This theory of T cell-mediated autoimmunity in SAA is further supported by the therapeutic potential of T cell immune suppression in the disease [2].

A significant proportion of cases of AA, at some point in their evolution, are associated with a paroxysmal nocturnal hemoglobinuria (PNH) clone. PNH is a clonal hematopoietic disorder characterized by a somatic mutation in the PIG-A gene that results in deficiency of complement protective glycosylphosphatidylinositol proteins CD55 and CD59 on the surface of blood cells. Complement-mediated intravascular hemolysis and thromboses are classical manifestations of PNH [3]. PNH can also evolve into other marrow disorders including AA, myelodysplasia, and acute myelogenous leukemia [4]. About 20 to 40% of patients with AA have a PNH clone at diagnosis. Late identification of a PNH clone following immunosuppressive therapy in AA has also been described [5–7]. Preferential survival of PNH clones in AA marrows has been attributed to relative resistance of the PNH clone to T cell cytotoxicity. Due to the decreased glycoprotein anchoring ability of the PNH cell, there is a decreased copy number of CD58 (lymphocyte function-associated antigen 3-ligand (LFA3)) on the red cell. Erythrocyte CD58 normally binds to CD2 and is responsible for T cell adhesion, activation, cytokine production and cytotoxic activity. CD58-depleted PNH cells are, therefore, relatively resistant to T cell-mediated effects, including autoimmunity [8, 9]. On the other hand, PNH clones may be more sensitive to B-cell suppression by the monoclonal anti-CD20 antibody rituximab. Preclinical data show that upregulation of the GPI anchor proteins may mediate resistance to rituximab, and the absence of these surface proteins in PNH may make the clone more susceptible to rituximab cytotoxicity [10]. Our case, to our knowledge, is the first of its kind to demonstrate an effect of rituximab in AA with a PNH clone.

## 2. Case Report

A 17-year-old Hispanic female at 12 weeks of pregnancy was referred in March 2007 for severe pancytopenia. She had presented with fatigue and shortness of breath during a routine prenatal visit. Her past medical history was unremarkable except for a first trimester miscarriage one year prior. She was taking prenatal vitamins and denied chemical exposure, smoking or alcohol use, or a family history of blood disorders. On exam there was severe conjunctival pallor but no scleral icterus. No lymphadenopathy or hepatosplenomegaly was palpable. Petechiae were noted on both lower extremities.

Complete blood counts showed pancytopenia: WBC: 3.8 × 10^9^/L; ANC: 1.4 × 10^9^/L; nadir ANC: 0.1 × 10^9^/L; Hb: 3.4 g/dL; platelets: 3 × 10^9^/L. Reticulocyte count was markedly low at 0.1% with an absolute reticulocyte count of 2.6 × 10^9^/L. Liver, renal function, and bilirubin were normal. Bone marrow biopsy showed an acellular marrow with 0 to 5% cellularity with marked reduction to absent trilineage hematopoiesis. Flow cytometry demonstrated <1% CD34^+^ CD117^+^ hematopoietic precursors. Bone marrow cytogenetics showed a normal female karyotype of 46XX; fluorescent in situ hybridization studies were negative for deletion 5q31, deletion 7q31, monosomy 5 and 7, trisomy 8 and 20q12 and p53 deletion. Serology revealed prior immunity to CMV, EBV, and parvovirus B-19. Hepatitis A, B, and C, HIV, and HTLV serologies were negative as was ANA and genetic testing for Fanconi's anemia. Serum B12 and folate levels were normal. SPEP and UPEP did not demonstrate a monoclonal protein. Flow cytometry for erythrocyte CD55 and CD59 was normal. A diagnosis of idiopathic very severe aplastic anemia (VSAA) was made. No sibling or unrelated donor could be identified as compatible for allogeneic bone marrow transplant (ABMT).

The patient's pregnancy was managed with transfusion support. At 32 weeks of gestation she presented in preterm labor with *Escherichia coli* sepsis. With prenatal steroids, antibiotics, and platelet support, she delivered a normal baby boy vaginally. In July 2007, 2 weeks after delivery, she received a course of intravenous equine antithymocyte globulin (h-ATG: 40 mg/kg daily for 4 days) and oral cyclosporine 12 mg/kg/d titrated to cyclosporine levels of 200 to 400 ng/mL. Neutrophils remained less than 1.0 × 10^9^/L, and she remained transfusion dependent. A second cycle of h-ATG and cyclosporine was given in October 2007, which was complicated by seizures at therapeutic cyclosporine levels, and the cyclosporine was stopped. Three months later, she still remained transfusion dependent with neutrophils less than 1.0 × 10^9^/L.

In January 2008, a course of rituximab 375 mg/m^2^ was administered weekly for four weeks. In March 2008 she demonstrated partial recovery of neutrophils with a neutrophil count consistently between 1 × 10^9^/L and 1.5 × 10^9^/L while remaining transfusion dependent for platelets and red cells. At this time she was hospitalized with abdominal pain due to ischemic bowel and Budd-Chiari syndrome. Repeat testing for PNH was positive demonstrating CD59 deficiency on 81% of the leukocytes with 54.7% deficiency on the granulocytes. In addition, CD59 was found to be deficient on 11.4% of the red cells. Reticulocyte count was still low at 0.1% with absolute reticulocyte count of 2.6 × 10^9^/L. Lactate dehydrogenase was elevated at 245 Units/mL. Attempts to anticoagulate her were unsuccessful due to persistent bleeding despite platelet transfusions. She underwent bowel resection for ischemic bowel and was discharged after a prolonged and complicated hospital course. In May 2008, ANC stabilized between 1 × 10^9^/L and 2 × 10^9^/L, but transfusion dependence for red cells and platelets persisted with nadir Hb of 5.5 gm/dL and platelets of 5 × 10^9^/L.

**Figure 1:**
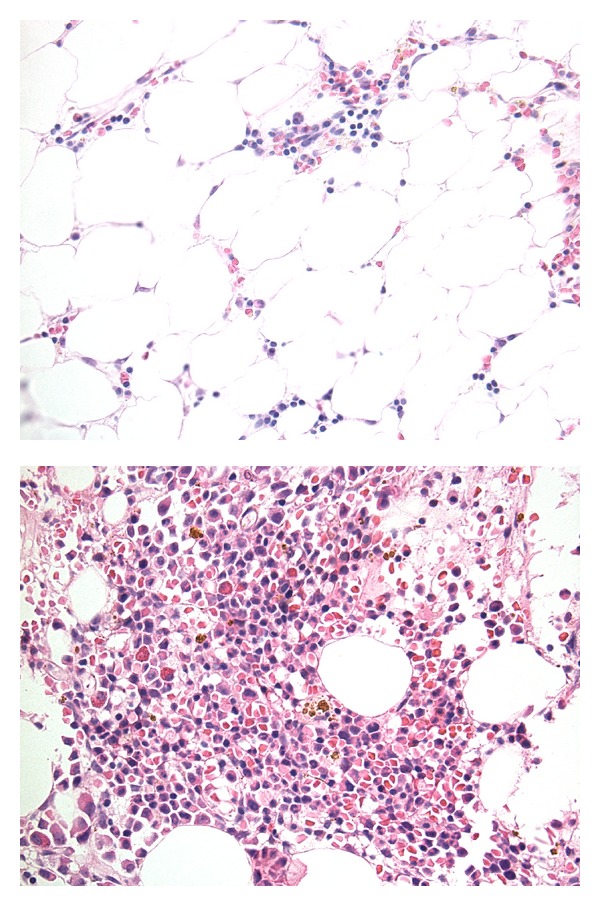
This figure illustrates the improvement in bone marrow biopsy findings with rituximab therapy. Pictures were taken under 40x magnification. The top figure shows the aplastic marrow on diagnosis in March 2007. The lower figure shows restoration of trilineage hematopoiesis with decreased megakaryocytes in July 2008, 6 months after the first cycle of rituximab.

**Figure 2:**
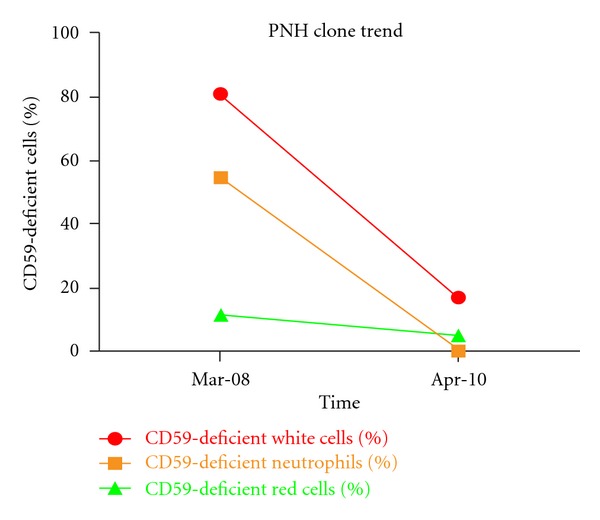
The reduction in PNH clone size over time. In March 2008, the CD59-deficient clone of WBCs and RBCs was detected when Budd-Chiari syndrome and intestinal ischemia were diagnosed. In April 2010, about 2 years after the first cycle of rituximab and 10 months after the second cycle of rituximab, there was significant decrease in size of the PNH clone in all cell lines.

**Figure 3:**
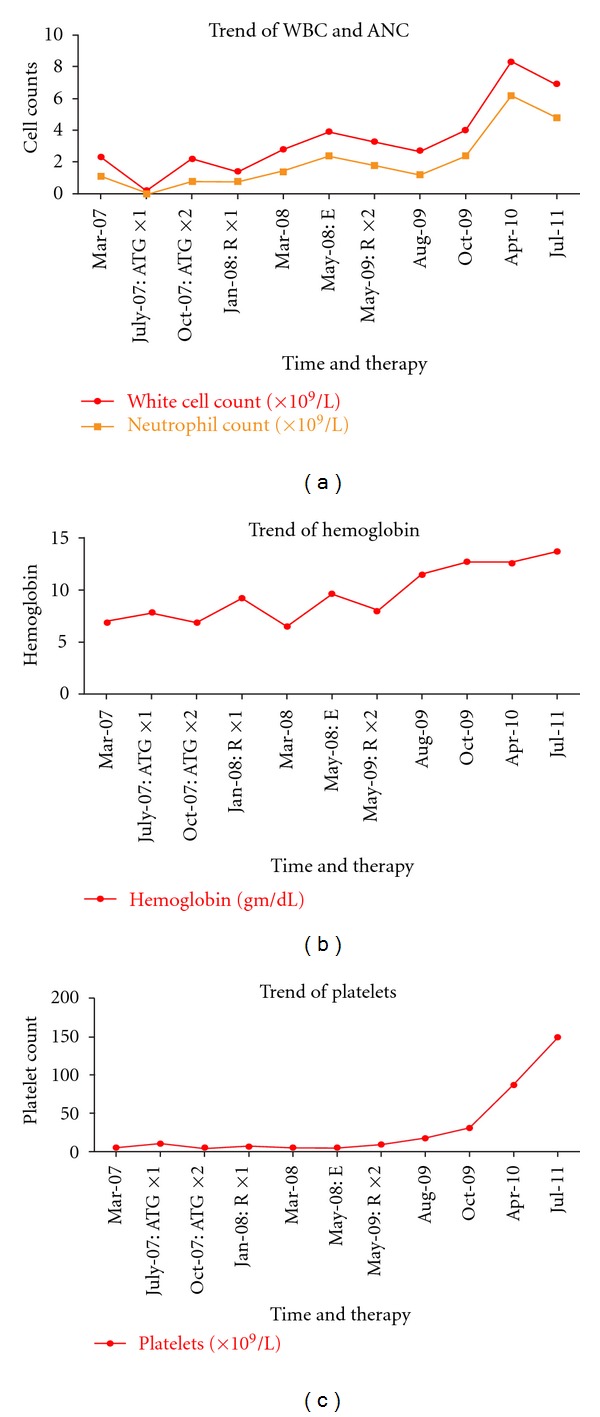
The improvement in blood counts from the time of diagnosis to most recent. (a) white cell and neutrophil counts, (b) describes the hemoglobin trend, and (c) the platelet trend. The *x* axis is not drawn to scale and shows various time points and therapy administered. ATG: antithymocyte globulin, R: rituximab, and E: eculizumab.

In July 2008, biweekly eculizumab was started with subsequent improvement in anemia and stabilization of hemoglobin at 9 gm/dL in May 2009. However platelet transfusions continued and neutrophils remained between 1 and 2 × 10^9^/L. Abdominal imaging showed resolution of the hepatic vein thromboses. No portosystemic collaterals, splenomegaly, or hepatic dysfunction had developed. Bone marrow biopsy showed a normocellular marrow (40%) with erythroid predominant hematopoiesis and decreased megakaryocytes (Figure 1). While thrombocytopenia could be partly explained by slow megakaryocyte recovery, whether peripheral destruction by persistent autoimmune mechanisms also contributed to ongoing pancytopenia is worth speculation. In June 2009, 17 months after the first cycle of rituximab, 4 additional doses of rituximab 375 mg/m^2^ weekly were given to facilitate platelet recovery. In October 2009, 4 months after the second course of rituximab, her hemoglobin normalized and her platelets began to recover. Repeat PNH testing in December 2009 and April 2010 showed significant decrease in the size of the PNH clone. In April 2010, only 17% of the leukocytes, 0% of the granulocytes, and 5% of the red cells were deficient in CD59 (Trend of CD59, deficient cells in Figure 2). In November 2011, 2.5 years after the second course of rituximab, the patient remained in a complete hematological response (CBC results shown in Figure 3). 

## 3. Discussion

To our knowledge this is the first case report showing efficacy of rituximab in reducing PNH clone size and facilitating restoration of hematopoiesis in PNH associated with AA. The earliest response of improved hematopoiesis with rituximab in idiopathic AA was seen 50 days after therapy while complete response took up to 16 months [11]. In our case, partial neutrophil recovery was seen as early as two months after rituximab, hemoglobin response occurred about 1 year after therapy, and while the platelets did not recover with the first course of rituximab they showed dramatic recovery 3 months after the second course. The initial improvement in neutrophils in March 2008 could be partly attributed to the delayed effect of T cell suppression. Hemoglobin recovery may have been facilitated by eculizumab; however, anemia in this case was predominantly hypoproliferative rather than hemolytic suggesting a significant role of rituximab in its reversal. The improvement of megakaryopoiesis in October 2009 clearly had a temporal correlation with the second course of rituximab therapy.

In our case, testing for a PNH clone on erythrocytes was negative on presentation with AA. We propose that the PNH clone may have been undetected due to initial testing of predominantly transfused red cells and failure to test the white cells. The PNH clone in peripheral blood may have become manifest with improvement in neutrophil count. Alternatively T cell immune suppression may have facilitated emergence of the resistant PNH clone. While detection of the PNH clone occurred 2 months after rituximab therapy, we hypothesize this was not a direct rituximab effect. The complete effect of rituximab on B-cell immunity as well as possibly the PNH clone is likely to have taken several months. PNH clone size declined significantly between March 2008 and April 2010, temporally correlating with rituximab effect. Additional information like immunoglobulin levels and B-cell quantification may have further supported this argument but was not obtained at that time.

PNH, an acquired hematopoietic stem cell disorder of the PIG-A gene, results in increased sensitivity of blood cells to complement-mediated destruction. Eculizumab, a humanized monoclonal antibody against C5, acts as a terminal complement inhibitor and is effective in targeting classical manifestations of PNH including complement-mediated hemolysis and thromboses [12]. However, eculizumab does not reduce PNH clone size or improve marrow failure in the disease [13]. ABMT is the only curative therapy for PNH that replaces the abnormal stem cell clone and improves hematopoiesis in concurrent AA. However, ABMT in PNH is limited by significant morbidity and mortality as well as donor availability, like in our case [14, 15].

PNH has a strong association with AA. Small to moderate PNH clones can be identified in a majority of patients with AA. In a retrospective analysis of 207 consecutive patients with severe AA, 40% of patients had a detectable PNH clone pretreatment and the median clone size was 9.7%. In about 30% of these patients with a detectable PNH clone, the clone size increased after IST. Development of persistent new PNH clones after IST was rare and occurred only in 10% of patients. Classic PNH manifestations of hemolysis and thromboses in patients with AA and a PNH clone was uncommon and was seen only in 7 patients, all of whom had a PNH clone size of >50% [16, 17]. Our patient demonstrates a rare case of a large PNH clone (81% CD59-deficient leukocytes and 54.7% deficient granulocytes) evolving after IST for AA and presenting with a classic PNH manifestation of Budd-Chiari syndrome. Management in such cases needs to be directed against both the marrow aplasia and the PNH clone causing thrombotic manifestations.

Marrow failure in idiopathic AA and AA associated with PNH appears to be immune mediated. Activation of the T cell immune response and autoimmune destruction of hematopoietic stem cells appears to pathogenic in most cases of AA and therefore T cell suppression has emerged as effective therapy [2]. Normal T cells are activated by exposure to antigens on the surface of antigen-presenting cells. When activated, T cells produce IL-2, which results in further proliferation of their counterparts. Cytokines IFN-*γ* and TNF-*α* are also produced by activated T cells, which result in upregulation of the Fas receptor causing apoptosis of target cells [18]. In patients with AA, often T cells are autoactivated resulting in excessive production of marrow toxic cytokines, IFN-*γ*, and TNF-*α*. Many theories have been proposed for T cell autoactivation in AA. Tbet, a T cell transcription factor, appears to be constitutively active in T cells of patients with AA, stimulating production of IFN-*γ* [19]. Polymorphisms in the cytokine encoding genes increasing synthesis of TNF-*α*, IFN-*γ*, and IL-6 and causing apoptosis of marrow progenitors have also been described [20, 21]. Perforin gene mutations may cause abnormal proliferation and activation of cytotoxic T cells in AA [22]. T cell immune suppression is therapeutic in 75% of cases of AA [23]. However, PNH clones appear to be resistant to the T cell mediated cytotoxicity as well as T cell immune suppression facilitating preferential survival of the PNH clone in AA marrows. This phenomenon is demonstrated in our case [16, 24, 25]. Preferential survival of PNH clones after T cell suppression may be particularly problematic in situations where a large PNH clone causes classical PNH manifestations superimposed on marrow failure, as in our patient. Alternative therapeutic strategies are required in these cases and B-cell suppression can be explored.

B-cell autoimmunity and production of multiple autoantibodies have recently been described to be pathogenic in AA, especially the antibody to Moesin that has been shown to increase production of marrow toxic cytokines through activating the ERK 1/2 pathway [11]. Case reports have described the efficacy of rituximab in therapy of idiopathic AA [11, 26]. Preclinical data also suggest that the absence of GPI-linked proteins may make the PNH clone exquisitely sensitive to rituximab-mediated cytotoxicity [10]. Our case report suggests that B-cell suppression by rituximab may be therapeutic for both marrow aplasia and the PNH clone.

AA is a uniformly fatal disease if left untreated, although rare cases of spontaneous remission have been described. Even with rapid institution of appropriate therapy, 10-year survival remains low. Median 10-year survival in AA is about 68% with immune suppressive therapy (IST) and 73% with ABMT [23]. More effective and less toxic therapies directed against the pathophysiology of AA are an urgent necessity to improve survival in this disease, especially since many afflicted patients are in their childhood and adolescence. In addition, management of a symptomatic large PNH clone in AA may be difficult, especially if a suitable ABMT donor is unavailable since the PNH clone is typically resistant to T cell immune suppression. Our case demonstrates that rituximab may be a very valuable drug with minimal toxicities in management of AA with a symptomatic large PNH clone. Larger clinical studies with correlative experiments will be required to validate our findings from this paper.
